# Multigene Identification of a Giant Wild Strain of *Ganoderma mutabile* (ZHM1939) and Screening of Its Culture Substrates

**DOI:** 10.3390/life15091475

**Published:** 2025-09-19

**Authors:** Huiming Zhou, Longqian Bao, Zeqin Peng, Yuying Bai, Qiqian Su, Longfeng Yu, Chunlian Ma, Jun He, Wanzhong Tan

**Affiliations:** College of Biotechnology and Engineering, West Yunnan University, Lincang 677000, China; gladhappy305@163.com (H.Z.);

**Keywords:** ZHM1939 strain, *Ganoderma mutabile*, morphology, multigene identification, phylogenic tree, optimized culture substrates

## Abstract

In the present study, a new *Ganoderma* sp. (ZHM1939) was collected from *Lincang*, Yunnan, China, and described on the basis of morphological characters and multigene phylogenetic analysis of rDNA-ITS, *TEF1α* and *RPB2* sequences. This fungus is characterized by the exceptionally large basidiomata, oval shape, a pileus measuring 63.86 cm long, 52.35 cm wide, and 21.63 cm thick, and a fresh weight of 80.51 kg. The skeleton hyphae from the basidiocarp are grayish to grayish-red in color, septate, and 1.41–2.75 μm in diameter, with frequently dichotomous branched and broadly ellipsoid basidiospores. The basidiospores are monocellular, ellipsoid, with round ends or one slightly pointed end, brown–gray in color, and measured 6.52–10.26 μm × 4.68–7.17 μm (*n* = 30). When cultured for 9 days at 25 ± 2 °C on PDA, the colony was white, ellipsoid or oval, with slightly ragged edges, measured Φ58.26 ± 3.05 mm (*n* = 5), and the growth rate = 6.47 mm/day; prosperous blast-spores formed after culturing for 21 days, making the colony surface powdery-white. The mycelia were septate, hyaline, branching at near-right angles, measured Φ1.28–3.32 μm (*n* = 30), and had some connections. The blast-spores were one-celled, elliptic or barley-seed shaped, and measured 6.52–10.26 μm × 4.68–7.17 μm (*n* = 30). Its rDNA-ITS, *TEF1α* and *RPB2* sequences amplified through PCR were 602 bp, 550 bp and 729 bp, respectively. Blast-n comparison with these sequences showed that ZHM1939 was 99.67–100% identical to related strains of *Ganoderma mutabile*. A maximum likelihood phylogenic tree using the concatenated sequence of rDNA-ITS, *TEF1α* and *RPB2* was constructed and it showed that ZHM1939 clustered on the same terminal branch of the phylogenic tree with the strains Cui1718 and YUAN 2289 of *G. mutabile* (Bootstrap support = 100%). ZHM1939 could grow on all the 15 original inoculum substrates tested, among which the best growth was shown on substrate 2 (cornmeal 40 g, sucrose 10 g, agar 20 g), with the fastest colony growth rate (6.79 mm/day). Of the five propagation substrates tested, substrate 1 (wheat grains 500 g, gypsum powder 6.5 g and calcium carbonate 2 g) resulted in the highest mycelium growth rate (7.78 mm/day). Among the six cultivation substrates tested, ZHM1939 grew best in substrate 2 (cottonseed hulls 75 g, rice bran 12 g, tree leaves 5 g, cornmeal 5 g, lime powder 1 g, sucrose 1 g and red soil 1 g) with a mycelium growth rate of 7.64 mm/day. In conclusion, ZHM1939 was identified as *Ganoderma mutabile*, which is a huge mushroom and rare medicinal macrofungus resource. The original inoculum substrate 9, propagation substrate 1 and cultivation substrate 2 were the most optimal substrates for producing the original propagation and cultivation inocula of this macrofungus. This is the first report on successful growing conditions for mycelial production, but basidiocarp production could not be achieved. The results of the present work establish a scientific foundation for further studies, resource protection and application development of *G. mutabile.*

## 1. Introduction

*Ganoderma* spp. are a group of globally distributed macrofungi [1] and have been widely documented across various geographical regions. While their distribution spans multiple climate zones, these fungi predominantly thrive in the warm areas of East Asia and subtropical areas due to their adaptation to hot and humid environments; they are able to grow in hot and humid environments [2]. Macrofungi (mushrooms) are of great edible, medicinal and economic value [3,4,5] and are utilized widely for medical treatments [6], in industry [7] and in food or food processing [8,9,10,11]. These macrofungi contain a variety of biochemical components beneficial to human health, which can inhibit tumor cell growth [11,12,13,14,15], improve immunity [16] and enhance memory [17]. Therefore, there is a long history of their application as foods and medicines [4,5]. However, very few species of *Ganoderma* have been cultivated and commercially utilized [18] although they are widely distributed and found in the wild.

Macrofungi are divided into types such as edible, medicinal, poisonous and wood-rotting fungi. According to literature reports, there are more than 10,000 macrofungal species distributed in China, among which there are nearly 1800 species of edible and 850 species of medicinal fungi [19]. Many different macrofungi have been found in Yunnan, which are distributed in all prefectures, cities and counties of the province [19], making Yunnan the most important province for edible and medicinal fungal resources in China. So far, the artificial cultivation and commercial production and application of domesticated macrofungi in China mainly includes edible mushrooms, such as *Lentinus edodes*, *Flammulina velutipes*, *Pleurotus ostreatus*, *Dictyophora indusiata*, *Auricularia auricula* and *Tremella fuciformis*, while research on the domestication [19,20] and application of other edible and medicinal fungi is less well developed. In particular, very few species of *Ganoderma* mushrooms have been successfully domesticated or commercially cultivated, which seriously hinders the sustainable development of the edible and medicinal mushroom industry and the use of most wild species. In the context of increasing destruction of the ecological environment of wild fungi and the lack of domesticated species [21], it is necessary to domesticate the cultivation of the present *Ganoderma* strain, as this will be of great importance to its successful cultivation and use.

*Ganoderma mutabile* was first recorded 10 years ago and was named as a new species by Cao & Yuan [22], but it has not been found or reported since then. This indicates that *G*. *mutabile* is a rare, precious resource of a medicinal mushroom species. In 2022, the authors found and collected an exceptionally large *Ganoderma* strain from rotten wood in *Wulao* Mountain coniferous forest in *Lincang* City, Yunnan Province, China. This strain was then morphologically described and molecularly identified. To establish a cultivation protocol for this rare species, a systematic substrate screening experiment was conducted. Based on this, fifteen original substrates (comprising five propagation and six cultivation inoculum substrates) were tested with general culture techniques [23] and the optimal substrates for in vitro mycelium growth of this wild *Ganoderma* were screened. This study identifies the optimal substrates for in vitro mycelial growth of *G. mutabile* and comprehensively reports the experimental methodology and outcomes. The results of these investigations are summarized and reported in this paper.

## 2. Materials and Methods

### 2.1. Materials

(1) The strain: The wild strain was collected from a rotten wood of coniferous forest on *Wulao* Mountain in *Lincang* City, Yunnan Province, China, in June 2022. Its basidiocarp is deposited in Fungarium Union of China (in *Hexi* University, *Zhangye* City, Gansu Province, China) with the code FHXU-ZHM1939. The pure culture of the strain is stored in the Mycology Laboratory of West Yunnan University, *Lincang*, China, coded ZHM1939.
(2) The pure culture: The pure cultures of ZHM1939 were obtained using tissue isolation and purification techniques. Mycelia from the pure cultures were used for microscopic observation of morphology and for extraction of genomic DNA [24].
(3) The PDA medium: The potato dextrose agar (PDA) medium was formulated with 200 g fresh peeled potatoes, 20 g dextrose and 20 g agar, plus tap water to 1000 mL. After being pasteurized at 121 °C (0.10–0.12 Mpa) for 30 min in an autoclave, PDA plates were made by aseptically pouring the medium into sterile Φ90 mm petri dishes (18 mL in each petri dish); PDA slants were made by pouring 10 mL into each of the 20 mL test tubes. The PDA plates and slants were used to culture and store the fungus, respectively.
(4) The primer pairs: The primer pairs ITS1/ITS4 [25], TEF1-983/TEF1-1567 [24] and RPB2-6f/fRPB2-7cR [26] were synthesized by *Suoqin* Biotech Co., Ltd., Beijing, China, and were applied to amplify the internal transcribed spacer (ITS), alpha 1 translation elongation factor gene (*TEF1α*) and RNA polymerase II second largest subunit gene (*RPB2*), respectively, through polymerase chain reaction (PCR).
(5) The culture substrates: The mycelium growth of ZHM1939 was measured on 15 original, 5 propagation and 6 cultivation inoculum substrates (Table 1) for screening the optimal substrates to prepare inocula to be employed at different stages of domestication and cultivation.

(6) Gene sequences downloaded: The ITS, *TEF1α* and *RPB2* gene sequences (Table 2) of 55 strains of related *Ganoderma* spp. were downloaded from the NCBI GenBank database and were used to construct a phylogenic tree for identification of the present ZHM1939 strain.

### 2.2. Methods

(1) Pure culture preparation: Initial cultures of ZHM1938 were isolated under aseptic conditions by transferring appropriately small amounts of internal tissues from the basidiocarp onto potato dextrose agar (PDA) medium and incubated at 25 ± 2 °C in darkness for 5 days. The tip mycelium from the edges of initial culture colonies was inoculated onto new PDA plates and incubated at 25 ± 2 °C and darkness for 9 days. Pure cultures of fungus were obtained by three repeated transfers with the same methods. After the PDA surface was fully covered with the fungal mycelium, the stock pure culture of the strain was deposited in the Applied Biology Laboratory of West Yunnan University, with the code ZHM1939. The cultures were maintained at 4 °C for further study.
(2) Morphological observations: These included macro-structural and microscopic observations.
  1. The macro-structural morphology. The basidiocarp and pure culture colonies of ZHM1939 were directly observed. For the basidiocarp, its total height (from the matrix to the pileus top), length and width diameters, and thickness of the pileus were measured; the shape and color changes of wild fresh and lab-dried samples were described and photographed. For the pure culture colony, its shape and color were observed and photographed; colonies of five replicates (in Φ90 mm petri dishes) were prepared and their diameters were measured with the crossing method [24] to obtain the mean value.
  2. The microscopic morphology. From the basidiocarp, an appropriately small amount of the tissue was picked up from its internal ventral surface with a sterilized needle, and the morphological characteristics of the skeleton hyphae and basidiospores were observed and 30 of each structure were measured under a microscope. The culture slant was activated at room temperature for 24 h, and an appropriate amount of mycelium was picked up from the slant, placed at the center of the PDA plates (in Φ90 mm petri dishes, 5 replicates), and incubated at 25 ± 2 °C in an incubator for 21 days. The morphology (shape, color etc.) of the colony was observed (photographed) at an appropriate time and the diameter of each colony (mm) was measured. At the same time, the morphological characteristics of the hyphae and spores (color, shape, septa, etc.) were observed under a microscope; the size of 50 spores and the diameter of the hyphae (μm) were measured.

According to the observed and recorded macro- and micro-characteristics, the taxonomic genus name of ZHM1939 was identified primarily by referring to Mao et al (2000) [4] and He et al (2022) [24].

(3) Molecular identification: The genomic DNA was extracted from the mycelium of ZHM1939 with the cetearyltrimethylammonium bromide (CTAB) method (White et al., 2021) [27]. The ITS, *TEF1α* and *RPB2* gene sequences were amplified from the DNA using the ITS1/ITS4, TEF1-983/TEF1-1567 and RPB2-6f/fRPB2-7cR primers. PCR amplification was performed following the method described by He et al. (2022) [24]. The PCR-amplified products were harvested and sequenced by Beijing *Suoqin* Biotech. Co. Ltd., China. The sequences were submitted to NCBI GenBank for registration of their accession numbers. Based on blast-n analyses of these sequences, ITS, *TEF1α* and *RPB2* sequences of related *Ganoderma* spp. were downloaded from NCBI GenBank (Table 2). Using the concatenated sequences of these *Ganoderma* spp. as an in-group and that of *Amauroderma rigosum* Cui 9011 strain as a contrast out-group, a maximum likelihood phylogenetic tree was constructed by RAxML-HPC2 on XSEDE on CIPRES Science Gateway [28]. The phylogenetic tree was visualized via Fig Treeversion 1.4.0 and edited with Power Point 2023 software. Finally, the species name of ZHM1939 was determined according to the phylogenetic tree.
(4) Preparation of substrates: The formulae of 15 original, 5 propagation and 6 cultivation inoculum substrates (Table 1) were designed and tested. Their preparation methods are briefly described as the follows.
  1. Original inoculum substrates. The materials of each substrate were fully mixed or dissolved in an appropriate amount of distilled water and then the volume of the medium was made to 1000 mL by adding distilled water. The solution of each medium was poured into 250 mL flasks and sterilized at 121 °C (0.10–0.12 Mpa) in an autoclave for 30 min. After cooling down to about 60 °C, the medium was poured into Φ90 mm petri dishes to make plates for use. The pH of substrate 7 was adjusted to pH6.5, and the pH of the other substrates was not adjusted. For substrates 1 to 7, the potatoes and/or carrots were peeled, washed and cut into about 1 cm^3^ cubes, and were boiled until being softened (but not rotten) and filtrated with double-layered gauze. The onion (in medium 13) was boiled with the same amount of water for 30 min and then filtered.
  2. Propagation inoculum substrates: These substrates were maintained at 65% of moisture and natural pH values. Each substrate was put into cylindrical glass bottles (Φ85 mm and height 118 mm); the bottles were filled fully with the substrate and sealed with moisture-proof paper. The bottled substrate was autoclaved at 121 °C (0.10–0.12 Mpa) for 90 min; after cooling down for 24 h, it was sterilized again at 121 °C (0.10–0.12 Mpa) for 60 min. The wheat grains of all the substrates were soaked in water for 24 h and then boiled until grains were softened; the grains were maintained in the pot for 5 min and the water was filtered from the wheat grains. The broad-leaf wood sawdust and pine needles (10–20 mm sections) were pre-soaked for 6 h and the red soil was sieved with a 200-mesh sieve before being used.
  3. Cultivation inoculum substrates: The substrates were maintained at about 65% of moisture and natural pH value. Each substrate was put into cylindrical polythene bags (Φ170 mm and height 330 mm); the bags were fully filled with the substrate and sealed with sticky paper. The substrate in the bags was autoclaved at 121 °C (0.14–0.14 Mpa) for 90 min; after cooling down for 24 h, it was sterilized again at 121 °C (0.10–0.12 Mpa) for 90 min. The wheat grains of all the substrates were soaked in water for 24 h and then boiled until 95% of the grains were softened; the grains were maintained in the pot for 5 min and the water was filtered from the wheat grains. The broad-leafed tree wood sawdust and pine needles (10–15 mm sections) were pre-soaked for 4–6 h and the red soil was sieved with a 200-mesh sieve before being used. The sawdust, cottonseed shells, corncobs, broad-leaf tree leaves, fungal bran, and broad bean shells were pre-soaked with tap water for 6–12 h; the cow-dung and red soil were crushed and sieved with a 200-mesh sieve; and the sugar was dissolved in an appropriate amount of tap water before it was were used.
(5) Test of ZHM1939 growth on different substrates: The growth test methods of ZHM1939 mycelium on original, propagation and cultivation inoculum substrates were as the follows:
  1. Test of original inoculum substrates: Φ5 mm mycelial discs were prepared by punching from the edges of pure culture colony of ZHM1939. One mycelial disc was placed at the center of a PDA plate and five plates were inoculated. The plates were then incubated at 25 ± 2 °C in darkness in an incubator; the moisture was that within the petri dishes (RH > 90%). The new colony on each PDA plate was observed and its diameter was measured 7 days post inoculation. The colony growth rate was calculated (mm/day): r (mm/day) = (mean radius − inoculated mycelia radius)/7.
  2. Test of propagation inoculum substrates: Φ10 mm mycelial discs were prepared by punching from the edges of the original inoculum colony of ZHM1939. Three of the mycelial discs were placed separately on the surface of each propagation inoculum substrate bottle. Each substrate treatment was replicated in five bottles each. The bottles were sealed and incubated at 25 ± 2 °C, under darkness and relatively high humidity, inside the bottles. The distance of mycelium growth between 5–15 days after inoculation was measured and the mean growth distance (D) of the 5 replicates was used for calculating the mycelium growth rate (mm/day): r = D/10.
  3. Test on cultivation inoculum substrates: Twenty grams of the ZHN1939-infested wheat grains from the propagation inoculum substrate was seeded uniformly on the surface of the cultivation inoculum substrate in each bag, and five bags were replicated for each substrate. The bags were sealed and incubated at 25 ± 2 °C under darkness and 65% humidity inside the culture bags. The distance of mycelium growth between 5–15 days after inoculation was measured and the mean growth distance (D) of the 5 replicates was used for calculating the mycelium growth rate (mm/day): r = D/10.

In all the above tests, the mycelium growth vigor was recorded as + (mycelium rare and week), ++ (mycelium moderate) or +++ (mycelium thick and strong) [29]. Duncan’s new multiple range test [30] was applied to compare the significance of differences between different media or substrates.

## 3. Results and Analysis

### 3.1. Morphological Characterization of ZHM1939

**Figure 1:**
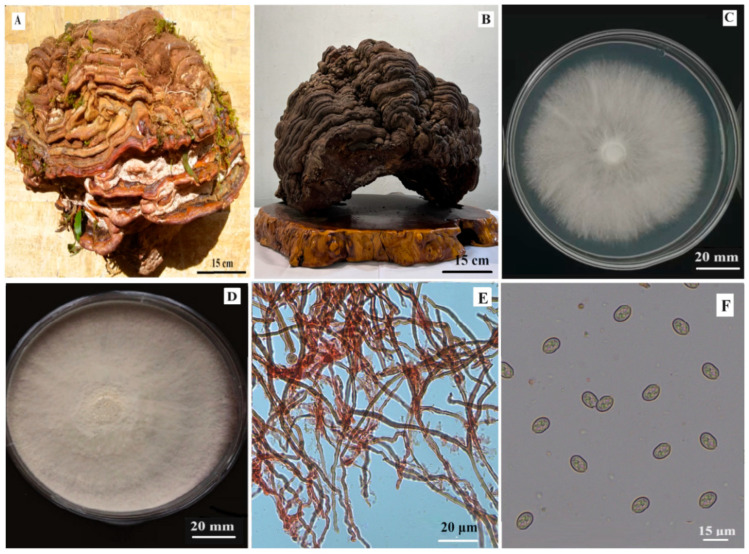
The morphological characteristics of *Ganoderma* sp. strain ZHM1939. Note: (**A**) Fresh wild basidiocarp. (**B**) Cleaned and dried basidiocarp maintained in fungarium for 12 months. (**C**,**D**) Colony of pure culture 9 and 18 days post inoculation. (**E**) Skeleton hyphae. (**F**) Basidiospores.

Macro-morphology: The fresh basidiocarp (Figure 1A) collected from the rotten coniferous wood of wild forest was exceptionally large. It was oval, coriaceous, corky to woody hard, and consisted clearly of 12 layers. The pileus surface was red–brown and measured 63.86 cm long, 52.35 cm wide and 21.63 cm thick, and the fresh basidocarp weighed 80.51 kg. Its younger tissue was glossless and the older tissue was paint-glossy. There were obvious concentric grooves and ring layers with smooth and rounded edges. Some white mycelial tissues were seen on the pileus surface and epiphytic mosses were growing in the grooves between the layers. After being cleaned and placed in the fungarium for 12 months, the fruiting body (Figure 1B) became shrunken and dark-brown. When cultured for 9 days at 25 ± 2 °C under darkness on PDA, the colony (Figure 1C) was white, round or oval, with slightly ragged edges, 58.26 ± 3.05 mm (*n* = 5) in diameter and with a growth rate = 6.47 mm/day. Prosperous blast-spores formed after being cultured for 18 days, making the colony surface turn powdery-white (Figure 1D).

Micro-morphology: Under the microscope, the hyphae from the pure culture colony were hyaline, septate, branched at near-right angles, 1.28–3.32 μm (*n* = 30) in diameter and had clear clamp connections. The blast-spores were one-celled, ellipse or barley-seed shaped, and 6.52–10.26 μm × 4.68–7.17 μm (*n* = 30). The skeleton hyphae (Figure 1E) from the basidiocarp were pale gray or red–gray, septate, 1.41–2.75 μm (*n* = 30) in diameter; some skeleton hypha tip sections were curved in spiral shapes. The basidiospores (Figure 1F) were ellipsoid, one-celled, deep gray, with rounded ends or with one slightly tapered end, and sized 6.52–10.26 μm × 4.68–7.17 μm (*n* = 50).

According to the above morphological characteristics of ZHM1939, it was primarily identified as *Ganoderma* sp. via referencing the related literature (Mao, 2000 [4]; He et al., 2022 [24]; Mardones et al., 2023 [31], Sun et al, 2022 [32]).

### 3.2. Molecular Identification of ZHM1939

**Figure 2:**
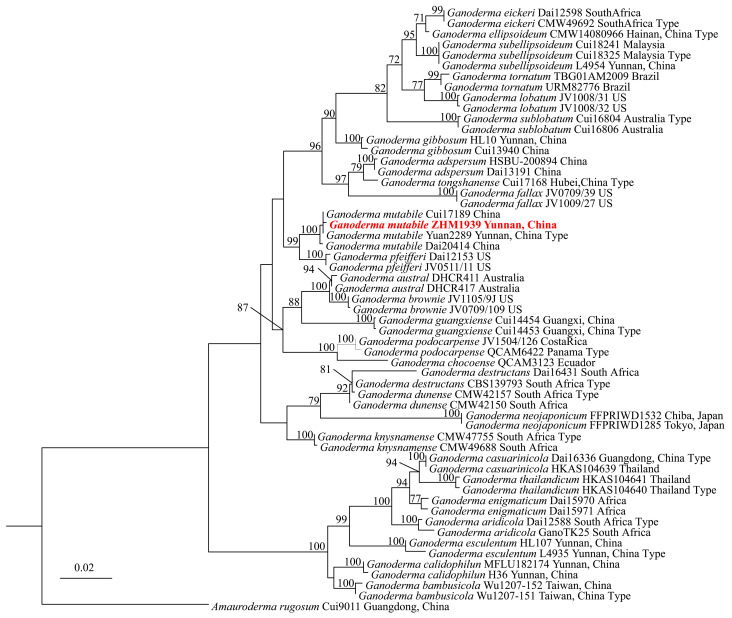
Maximum likelihood phylogenetic tree of *Ganoderma* spp. Note: The tree is constructed based on the concatenated sequence of ITS, *TEF1α* and *RPB2*. The number at each node shows Bootstrap confidence of the paired organisms of being the same species and only the branches with Bootstrap support >70 are shown. The present Ganoderma strain ZHM1939 is shown in red color.

The genomic DNA of ZHM1939 was extracted from the mycelia of ZHM1939 pure culture, and the ITS, *TEF1α* and *RPB2* sequences PCR-amplified were 632 bp, 550 bp and 729 bp, respectively. The sequences were deposited in NCBI GenBank under accession numbers of PV545123, PV550967 and PV550966, respectively; blast-n comparison analyses showed that ZHM1939 was 99.67–100% identical to *G. mutabile*. Based on the results of the blast-n analyses, the ITS1, *TEF1α* and *RPB2* sequences from strains of related *Ganoderma* spp. were downloaded (Table 2) from the GenBank database and were concatenated. A maximum likelihood phylogenetic tree (Figure 2) based on concatenated sequences was constructed and it was shown that ZHM1939 and *G. mutabile* strains Cui17189 and Yuan2289 were claded together on the same terminal branch with Bootstrap support of 100%. The results of the phylogenetic analysis of the DNA sequences were in good agreement with that of the blast-n comparisons. Consequently, the ZHM1939 strain was identified as *G. mutabile*.

### 3.3. Growth of ZHM1939 on Original, Propagation and Cultivation Inoculum Substrates

**Figure 3:**
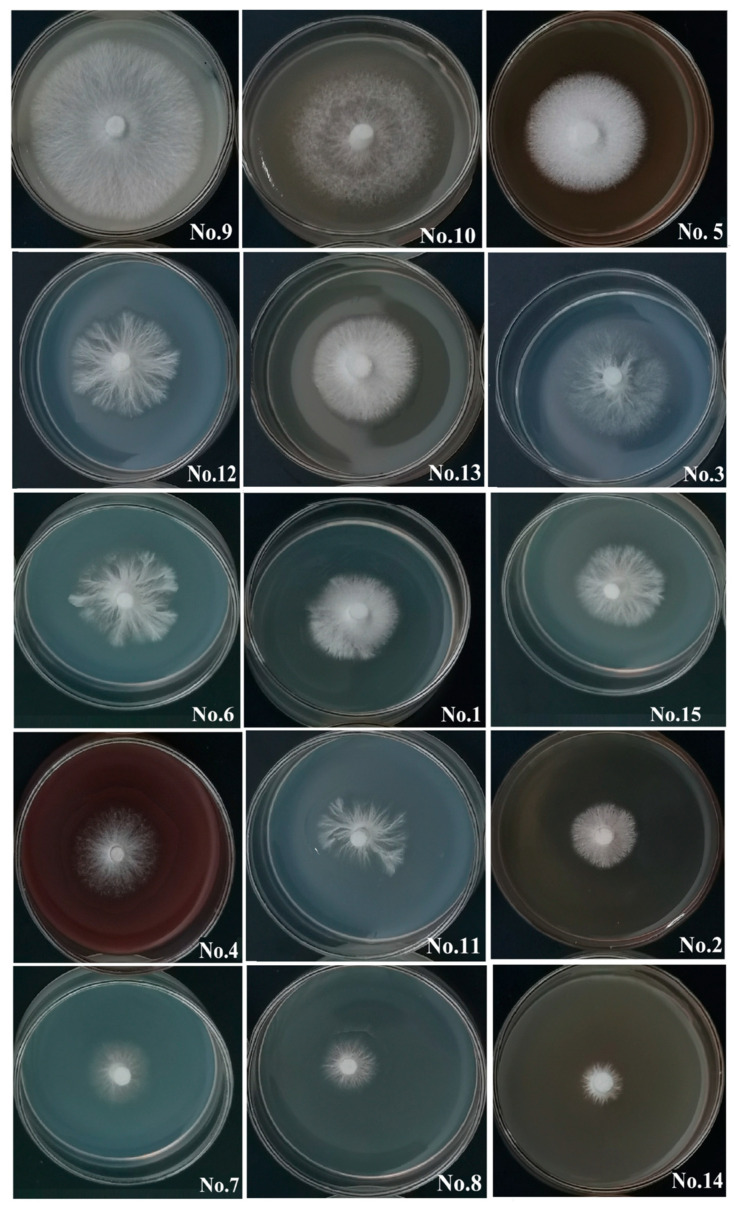
Colony growth on the 15 original inoculum substrates tested. Note: The code numbers refer to different media (refer to Table 1); photos were taken 7 days after treatment and incubation under 25 ± 2 °C in darkness; the plates in the figure are organized based on the colony diameter in descending order.

(1) Growth on original inoculum substrates: A number of 15 original inoculum substrates were tested and the results of ZHM1939 colony growth are presented in Table 3 and visually shown by Figure 3. ZHM1939 produced white colonies on all the substrates tested, but the colonies varied apparently in morphology and size, resulting in significantly different growth rates (*p* < 0.01) between those on various substrates. After culture at 25 °C on substrate 9 (corn meal 40 g, sucrose10 g, agar 20 g) for 7 days, the colony was the largest (Φ47.51 ± 2.42 mm) and its growth rate was calculated as 6.79 mm/day; the mycelium was the most condensed and robust (Figure 3). Contrarily, the colony (Φ13.32 ± 1.15 mm) formed on substrate 14 (apple juice 200 mL and agar 20 g) was the smallest, with the slowest growth rate (1.91 mm/day); the mycelium of the colony was relatively weak and scarce (Figure 3). Therefore, substrate 9 was the best for producing the original inoculum of ZHM1939.

(2) Growth on propagation inoculum substrates: Mycelium growth in five different propagation inoculum substrates was observed and the results are presented in Table 4. These results indicate that the mycelium was able to grow to fill bottles of all the propagation inoculum substrates, but the time needed varied from 18.63 days (substrate 1) to 23.42 days (substrate 4). Accordingly, mycelial growth and growth rates were significantly different between different substrates (*p* < 0.01). The best one was cultivation substrate 1 (wheat grains 500 g, gypsum powder 6.5 g and calcium carbonate 2.0 g), in which the mycelium was most condensed and its growth rate was the highest (7.78 mm/dayay) among the five tested cultivation substrates. In cultivation substrate 2, the mycelium was also rather condensed and vigorous and grew fast with a growth rate of 6.65 mm/dayay. In substrate 4, however, the mycelium grew most slowly (growth rate = 4.02 mm/dayay) and much less densely. Consequently, substrate 1 was the best propagation inoculum substrate for culturing ZHM1939.

(3) Growth on cultivation inoculum substrates: In Table 5, the results of the ZHM1939 mycelium growth in the 6 cultivation inoculum substrates tested are shown. Apparently, mycelium growth in cultivation substrate 2 was the fastest (7.64 mm/day) and most vigorous among all the tested substrates, whereas it was the slowest (4.43 mm/day) and was much less exuberant in cultivation substrate 6. As a result, the time needed for the mycelium to grow to the full cultivation bag was nearly 11 days less in substrate 2 (37.12 d) than in substrate 6 (48.03 days). Therefore, substrate 2 was the best cultivation substrate for growing ZHM1939.

## 4. Discussion

*Ganoderma mutabile* Y Cao & S H Yuan was a new species of *Ganoderma* that was first named before the year 2013 and it its specimens collected from living angiosperm trees in *Zixi* Mountain Nature Reserve, *Chuxiong* of Yunnan Province, China, and preserved in the Applied Ecology Laboratory of Chinese Academy of Sciences, Beijing, China (specimen voucher No. 8.IX.2006 Yuan 2289). The pileus of species was 7.5 cm high, 11 cm wide and 2.7 cm thick [22]. In June 2022, we collected a giant wild *Ganoderma* strain on the rotten wood of coniferous forest in *Wulao* Mountain Forest in *Linxiang* District, *Lincang*, Yunnan Province, and its basidiocarp was deposited in Fungarium Union of China (in *Hexi* University, Gansu Prov., China) with the code FHXU-ZHM1939, which was identified as *G. mutabile* in the present study. However, there are great differences between the fruiting body of the ZHM1939 strain reported here and those of the *G. mutabile* strain reported by Cao and Yuan [22]. The basidiocarp of ZHM1939 is as high as 31.56 cm, its pileus is oval, sized 63.86 cm × 52.35 cm × 21.63 cm, and it has a fresh weight of 80.51 kg, illustrating that ZHM1939 is an exceptionally giant *Ganoderma* strain. The difference between the fruiting body sizes of the two strains are probably due to their different living environments and ages. Yuan 2289 strain was collected from living angiosperm trees in *Chuxiong* and it is an annual or biannual mushroom; the nutrients available on the living trees are much less abundant or accessible than those *Ganoderma* strains of rotten tree woods. However, ZHM1939 was found on a rotten wood matrix of coniferous forest in *Lincang*, China, and it is estimated to have grown there for more than 10 years. It can be seen that the fruiting body sizes of the same *Ganoderma* species are extremely variable in different natural wild forest environments. Exploring and understanding the relationship between the variation in *G. lucidum* strains and the living environments presents important implications for the domestication and cultivation of this species.

The combined use of morphology and multigene molecular biology is a strategy widely employed by scholars in recent years for identifying fungal species (Sun et al, 2022 [32]; Yang et al, 2022 [33]; Yuan et al, 2023 [34]; Xing, 2019 [1]; Xing et al, 2018 [35]). Based on the description of the morphological characteristics and the results of polygenic analysis with the ITS, *TEF1α* and *RPB2* sequences, ZHM1939 was identified as *G. mutabile*. In the recent three-domain classification system of cellular organisms (Woese et al., 1977 [36]; Adl et al., 2019 [37]; Shen et al., 2019 [38]), *G. mutabile* belongs to the domain Eukarya, kingdom Fungi, phylum Basidiomycota, class Hymenomycetes, order Polyporales, family Ganodermaceae and genus Ganoderma. ZHM1939 is the only specimen of this species collected after more than 10 years of the primary publication of *G. mutabile* by Cao & Yuan [22], indicating that *G. mutabile* is a very rare species of the Ganoderma genus in the wild. Also, the present identification was confirmed based on the morphology of one basidiocarp and its mycelial cultures. Further surveys and investigations are necessary to understand the distribution and abundance of the species, and thus to effectively protect and utilize its resources.

Cultivatable edible and medicinal fungi generally use rotten woods or other decaying plant materials as their growing substrates, and their artificial cultivation is often based mainly on different plant decaying substrates with high lignin and cellulose contents [39]. It was observed that the ZHM1939 strain of *G. mutabile* was also a wood-rotten fungus when it was first found and its specimen was collected. Based on the identification of its species, this study tested its growth potential on different culture substrates. The results showed that the growth rates and growth potential of ZHM1939 on various tested substrates were significantly different (*p* < 0.01), so the best substrates suitable for its growth were optimized and screened out. The component materials of the cultivation inoculum substrate optimized here for ZHM1939 were different from those screened for producing cultivation inocula of other *Ganoderma* spp. reported previously by Chen et al. (2023) [40] and Wannasawang et al. (2023) [41]. This may be mainly because the nutritional components required by different *Ganoderma* species or strains are not completely the same. Our results can be referenced for the domestication and cultivation of ZHM1939.

Based on the cultivation inoculum substrate screening results in this study, a preliminary soil cultivation experiment of ZHM1939 was carried out in the laboratory with the selected optimal cultivation substrate. The mycelium growth was observed to be very vigorous in the mixture of the optimal substrate and soils in pots, but unfortunately badiocarp production was not achieved; no mycelium differentiation and fruiting body formation was observed until 60 days of sowing the cultivation inoculum. In this regard, we will continue to carry out further experiments to clarify the nutritional and other cultivation conditions (temperature, humidity, lighting regimes, pH values etc.) required for ZHM1939 to form fruiting bodies.

After being identified, the strain, together with its mycelium cultivation method, was patented in China with patent number CN119242454 (Zhou et al, 2025 [42]).

## 5. Conclusions

To summarize from the results, ZHM1939 is a giant wild *Ganoderma* and it is identified as *G. mutabile*. The culture substrates influenced the growth of *G. mutabile*. Among them, the original inoculum substrate 9 (corn flour 40 g, sucrose 10 g and agar 20 g), the propagation inoculum substrate 1 (wheat flour 500 g, gypsum 6.5 g, calcium carbonate 2 g), and the cultivation inoculum substrate 2 (cottonseed shell 75 g, rice bran 12 g, leaves 5 g, corn meal 5 g, lime 1 g, sucrose 1 g, red soil 1 g) were the best substrates for producing the three levels of inocula of ZHM1939. Further studies of *G. mutabile* on the physical and biological factors affecting its vegetative growth, basidiocarp production, and functional biochemicals of medicinal significance are necessary. The results of the present work have established a scientific foundation for further studies, resource protection, and application of *G. mutabile* ZHM1939.

## 6. Patent

Huiming Zhou et al. A strain of *Ganoderma mutabile* and method of its mycelium cultivation, Patent No. ZL2024 1 1285569.7, China National Intellectual Property Administration, 15 April 2025.

## Figures and Tables

**Table 1:** Formula of original, propagation and cultivation inoculum substrates tested.

Substrate for	Number	Medium Components
Original inoculum	1	Potato 200 g, dextrose 20 g
	2	Potato 200 g, sucrose 20 g
	3	Potato 200 g, maltose 20 g
	4	Potato 200 g, sucrose 20 g, peptone 10 g
	5	Potato 200 g, sucrose 20 g, Vitamin B1 1 mg mono potassium phosphate 3 g, magnesium sulfate 1.5 g
	6	Potato 200 g, carrot 80 g
	7	Sucrose 20 g, peptone 2 g, mono potassium phosphate 0.5 g, magnesium sulfate 0.5 g (pH 6.5)
	8	Sucrose 1 g, peptone 10 g
	9	Yeast powder 5 g, sucrose 20 g
	10	Sucrose 20 g, rice bran 50 g, monopotassium phosphate 0.3 g, mono potassium phosphate 0.2 g
	11	Sucrose 20 g, peptone 2 g, Vitamin B1 0.05 mg, monopotassium phosphate 0.46 g, mono potassium phosphate 0.5 g
	12	Sorghum powder 30 g
	13	Sucrose 50 g, onion 100 g, soy sauce 50 mL
	14	Apple juice 200 mL, agar 20 g (pH 6.4–6.8)
	15	Sucrose 20 g, peptone 2 g, yeast powder 2 g
Propagation inoculum	1	Wheat grains 500 g, gypsum powder 6.5 g, calcium carbonate 2 g
	2	Formula 1 + broad-leaf tree sawdust 25 g
	3	Formula 1 + red soil powder 25 g
	4	Formula 1 + wheat bran 25 g
	5	Formula 1 +, pine leaves 25 g
Cultivation inoculum	1	Wood saw dust 75 g, rice bran 12 g, tree leaves 5 g, corn meal 5 g, gypsum powder 1 g, sugar 1 g, red soil 1 g
	2	Rice bran 12 g, tree leaves 5 g, corn meal 5 g, gypsum powder 1 g, sugar 1 g, red soil 1 g, cottonseed hulls 75 g
	3	Rice bran 12 g, tree leaves 5 g, corn meal 5 g, gypsum powder 1 g, sugar 1 g, red soil 1 g, corncob 75 g
	4	Rice bran 12 g, tree leaves 5 g, corn meal 5 g, gypsum powder 1 g, sugar 1 g, red soil 1 g, corncob 60 g, mushroom bran 15 g
	5	Rice bran 12 g, tree leaves 5 g, corn meal 5 g, gypsum powder 1 g, sugar 1 g, red soil 1 g, corncob 45 g, mushroom bran 15 g, broad bean skin 15 g
	6	Rice bran 12 g, tree leaves 5 g, corn meal 5 g, gypsum powder 1 g, sugar 1 g, red soil 1 g, corncob 30 g, mushroom bran 15 g, broad bean skin 15 g, dried cow dung 15 g

Note: See the Methods section below for details for preparation methods of different substrates. Here, 20 g of agar was added to each of the original substrates. The pH values are not adjusted for the substrates except where it is notified.

**Table 2:** Accession numbers of ITS, *TEF1α* and *RPB2* sequences from strains of related *Ganoderma* spp.

Species	Voucher/Strain	Origin	GenBank Accession Numbers		
			ITS	*TEF 1α*	RPB2
*G. adspersum*	HSBU200894	China	MG279154	MG367542	-
*G. adspersum*	Dai13191	China	MG279153	MG367541	MG367492
*G. aridicola*	Dai12588T	South Africa	KU572491	KU572502	-
*G. aridicola*	GanoTK25	South Africa	JN105708	-	-
*G. australe*	DHCR411	Australia	MF436675	MF436677	-
*G. australe*	DHCR417	Australia	MF436676	MF436678	-
*G. bambusicola*	Wu1207-151T	Taiwan, China	MN957781	LC517941	LC517944
*G. bambusicola*	Wu1207-152	Taiwan, China	MN957782	LC517942	LC517945
*G. brownii*	JV0709/109	US	KF605662	MG367548	MG367495
*G. brownii*	JV1105/9J	US	MG279159	MG367547	MG367494
*G. calidophilun*	MFLU182174	Yunnan, China	MN398337	-	-
*G. calidophilun*	H36	Yunnan, China	MW750241	MW838997	MW839003
*G. casuarinicola*	HKAS104639	Thailand	MK817650	MK871328	MK840868
*G. casuarinicola*	Dai16336 T	Cantong, China	MG279173	MG367565	MG367508
*G. chocoense*	QCAM 3123	Ecuador	MH890527	-	-
*G. destructans*	CBS139793 T	South Africa	NR132919	-	-
*G. destructans*	Dai16431	South Africa	MG279177	MG367569	MG367512
*G. dunense*	CMW42150	SouthAfrica	MG020249	MG020228	-
*G. dunense*	CMW42157 T	South Africa	MG020255	MG020227	-
*G. eickeri*	CMW49692 T	South Africa	MH571690	MH567287	
*G. eickeri*	Dai 12598	South Africa	MZ354965	-	-
*G. ellipsoideum*	CMW14080966	Hainan, Chin	MH106867	MZ221652	-
*G. ellipsoideum*	L4954	Yunnan, China	ON994242	OP508446	-
*G. enigmaticum*	Dai15971	Africa	KU572487	KU572497	MG367514
*G. enigmaticum*	Dai15970	Africa	KU572486	KU572496	MG367513
*G.esculentum*	L4935T	Yunnan, China	MW750242	MW838998	MW839004
*G.esculentum*	HL107	Yunnan, China	ON994243	OP508437	OP508424
*G. fallax*	JV1009/27	US	KF605655	-	-
*G. fallax*	JV0709/39	US	KF605658	-	-
*G. gibbosum*	HL10	Yunnan, China	ON994245	OP508434	OP508421
*G. gibbosum*	Cui13940	China	MZ354972	MZ221658	MZ245404
*G. guangxiense*	Cui14453T	Guangxi, China	MZ354939	MZ221661	MZ245407
*G. guangxiense*	Cui14454	Guangxi, China	MZ354941	MZ221662	MZ245408
*G. knysnamense*	CMW47755T	South Africa	MH571681	MH567261	
*G. knysnamense*	CMW49688	South Africa	MH571684	MH567274	
*G. lobatum*	JV1008/31	US	KF605671	MG367553	MG367499
*G. lobatum*	JV1008/32	US	KF605670	MG367554	MG367500
*G. mutabile*	Yuan 2289T	Yunnan, China	JN383977	-	-
*G. mutabile*	Cui17189	China	MZ354976	MZ221679	-
*G. mutabile*	Dai20414	China	MZ354977	MZ221680	MZ345735
***G. mutabile ***	**ZHM1939**	**Yunnan, China **	**PV545123**	**PV550967**	**PV550966**
*G. neojaponicum*	FFPRI WD1532	Chiba, Japan	MN957785	-	-
*G. neojaponicum*	FFPRI WD1285	Tokyo, Japan	MN957784	-	-
*G. pfeifferi*	JV0511/11	US	KF605660	-	-
*G. pfeifferi*	Dai12153	US	MG279164	MG367559	
*G. podocarpense*	QCAM 6422T	Panama	MF796661	-	-
*G. podocarpense*	JV1504/126	Costa Rica	MZ354942	MZ221687	MZ345737
*G. subellipsoideum*	Cui18325T	Malaysia	-	MZ221702	-
*G. subellipsoideum*	Cui18241	Malaysia	-	MZ221701	-
*G. sublobatum*	Cui16804T	Australia	MZ354973	MZ221704	MZ345747
*G. sublobatum*	Cui16806	Australia	MZ354974	MZ221705	-
*G. thailandicum*	HKAS104640 T	Thailand	MK848681	MK875829	MK875831
*G. thailandicum*	HKAS104641	Thailand	MK848682	MK875830	MK875832
*G. tongshanense*	Cui17168T	Hubei, China	MZ354975	MZ221706	-
*G. tornatum*	TBG01AM2009	Brazil	JQ514108	-	-
*G. tornatum*	URM82776	Brazil	JQ514110	-	-
*Amauroderma rugosum*	Cui9011	Kantong, China	KJ531664	KU572504	MG367506

Note: The accession numbers of the DNA sequences were from the NCBI GenBank database. The accession numbers of ZHM1939 sequences are shown in red and boldface.

**Table 3:** Growth of ZHM1939 on different original inoculum substrates tested.

Substrate No.	Colony Diameter (mm)	Growth Rate (mm/day)	Growth Vigorousness
9	47.51 ± 2.42 A	3.04 ± 0.06 A	+++
10	39.92 ± 0.91 B	2.49 ± 0.13 B	+++
5	31.67 ± 2.31 C	1.90 ± 0.33 C	++
12	31.27 ± 2.53 C	1.90 ± 0.08 C	++
13	27.76 ± 1.84 CD	1.62 ± 0.08 CD	++
3	27.58 ± 1.12 CD	1.61 ± 0.16 CD	++
6	26.67 ± 1.33 DE	1.55 ± 0.19 DE	++
1	25.65 ± 1.19 DE	1.48 ± 0.17 DE	++
15	23.09 ± 1.47 DEF	1.29 ± 0.21 DEF	++
4	22.33 ± 0.56 EF	1.23 ± 0.08 EF	+
11	22.08 ± 0.49 EF	1.22 ± 0.07 EF	+
2	21.92 ± 2.03 EF	1.21 ± 0.29 EF	+
7	20.65 ± 1.05 F	1.12 ± 0.15 F	+
8	18.58 ± 0.21 F	0.97 ± 0.03 F	+
14	13.32 ± 1.15 G	0.60 ± 0.04 G	+

Note: (1) The colony diameter was measured 7 days after incubating ZHM1939 at 25 ± 2 °C on PDA plates. Each number is the mean of 5 replicates ± standard deviation. The capital letters indicate that there exist very significant (*p* < 0.01) differences between colony diameters and growth rates on different media. (2) The colony growth rate (mm/dayay) r (mm/dayay) = (mean radius − inoculated mycelia radius)/7. (3) The +, ++ and +++ in the growth vigorousness column indicate mycelium is sparse, denser and dense, respectively, in the colony observed 7 days after inoculation.

**Table 4:** Growth of ZHM1939 in different propagation inoculum substrates tested.

Substrate	Colony Full Time (d)	Colony Distance (mm)	Grow. Rate (mm/day)	Grow. Vigorousness
1	18.63 ± 0.82 D	77.82 ± 2.15 A	7.78 ± 0.21 A	++
2	21.02 ± 2.25 B	66.50 ± 2.82 B	6.65 ± 0.28 B	++
5	20.38 ± 2.15 BC	22.17 ± 1.26 D	1.12 ± 0.13 D	++
3	22.50 ± 1.36 AB	44.83 ± 2.84 C	4.28 ± 0.28 C	+
4	23.42 ± 3.20 A	40.21 ± 2.13 C	4.00 ± 0.21 C	+

Note: (1) The number of days that a colony grows to a substrate bottom from the surface is the mean ± standard deviation of five replicates. (2) The colony growth distance (D) is the distance that the mycelium grows in 10 days and each number is the mean ± standard deviation of five replicates. (3) Different capital letters after numbers indicate significant differences between substrates at *p* < 0.01. (4) The growth rate (mm/dayay) = D/10. 4). The +, and ++ in the growth vigorousness column indicate the mycelium is moderate and dense, respectively, in the substrates.

**Table 5:** Growth of ZHM1939 on different cultivation inoculum substrates tested.

Substrate	Colony Full Time (d)	Colony Growth (mm)	Growth Rate (mm/day)	Growth Vigorousness
2	37.12 ± 1.92 CD	76.63 ± 2.08 A	7.64	++
4	39.21 ± 2.04 C	63.32 ± 1.49 B	6.31	++
3	41.16 ± 2.25 BC	61.71 ± 1.92 B	6.16	++
1	43.96 ± 2.85 B	54.47 ± 1.23 C	5.61	++
5	46.87 ± 3.26 A	45.38 ± 0.74 D	4.54	+
6	48.03 ± 2.92 A	44.36 ± 0.91 D	4.43	+

Note: (1) The number of days that mycelium grows to substrate bottom from the surface is the mean ± standard deviation of 5 replicates. (2) The colony growth distance (D) is the distance that the mycelium grows in 10 days and each number is the mean ± standard deviation of five replicates. (3) Different capital letters after numbers indicate significant difference between substrates at *p* < 0.01. (4) The growth rate (mm/day) = D/10. (5). The + and ++ in the growth vigorousness column indicate the mycelium is moderate and dense, respectively, in the substrates.

## Data Availability

The original contributions presented in this study are included in the article; further inquiries can be directed to the corresponding author.
